# Design of an Aluminum/Polymer Plasmonic 2D Crystal for Label-Free Optical Biosensing

**DOI:** 10.3390/s18103335

**Published:** 2018-10-05

**Authors:** Luca Tramarin, Carlos Angulo Barrios

**Affiliations:** 1 Instituto de Sistemas Optoelectrónicos y Microtecnología (ISOM), ETSI Telecomunicación, Universidad Politécnica de Madrid, Ciudad Universitaria s/n, 28040 Madrid, Spain; luca.tramarin@alumnos.upm.es; 2 Department of Photonics and Bioengineering (TFB), ETSI Telecomunicación, Universidad Politécnica de Madrid, Ciudad Universitaria s/n, 28040 Madrid, Spain

**Keywords:** label-free optical biosensor, plasmon resonance biosensor, nanostructured surface, aluminum, design optimization, photonic modeling

## Abstract

A design study of a nanostructured two-dimensional plasmonic crystal based on aluminum and polymeric material for label-free optical biosensing is presented. The structure is formed of Al nanohole and nanodisk array layers physically separated by a polymeric film. The photonic configuration was analyzed through finite-difference time-domain (FDTD) simulations. The calculated spectral reflectance of the device exhibits a surface plasmon polariton (SPP) resonance feature sensitive to the presence of a modeled biolayer adhered onto the metal surfaces. Simulations also reveal that the Al disks suppress an undesired SPP resonance, improving the device performance in terms of resolution as compared to that of a similar configuration without Al disks. On the basis of manufacturability issues, nanohole diameter and depth were considered as design parameters, and a multi-objective optimization process was employed to determine the optimum dimensional values from both performance and fabrication points of view. The effect of Al oxidation, which is expected to occur in an actual device, was also studied.

## 1. Introduction

Plasmonic devices based on metal nanostructures can concentrate and enhance light in their near surroundings, which may be exploited to increase light–matter interaction in nanoscale spatial regions [1]. This remarkable property has enabled the development of highly sensitive label-free optical biosensors [2,3] based on either the sensitivity of surface plasmon polariton (SPP) and/or localized surface plasmon (LSP) resonances to changes of the refractive index at metal/dielectric interfaces, or Raman signal amplification due to SP electromagnetic field enhancement (surface-enhanced Raman spectroscopy). These plasmonic biosensing structures typically include metal nanoparticles in colloidal solutions [4,5,6] and periodic arrangements of metal nanostructures on planar substrates [7,8,9,10,11,12]. Colloidal metal nanoparticles offer important advantages, such as simple and cost-effective synthesis by bottom-up approaches; however, the creation of well-defined nanopatterns on planar substrates for chip-based applications is challenging. This can be achieved by employing common nanolithography techniques, such as nanoimprinting, electron-beam lithography, and focused ion beam, which are particularly well suited for parallelization on-a-chip and integration within microfluidic platforms [13,14,15,16] on a variety of support materials [17,18].

Hitherto, most label-free plasmonic biosensors have been developed based on Au due to its chemical inertness and good plasmonic characteristics in the visible spectrum. Apart from Ag, other metals have been scarcely studied for biosensing applications. One of these alternative materials, Al, is particularly appealing. Although Al has more optical losses than Au and Ag in the visible spectrum (e.g., the extinction coefficients of Al, Au, and Ag at 500 nm wavelength are 6.05, 1.87, and 3.13, respectively [19]), it presents important advantages such as low cost (Al is approximately 25,000 and 425 times cheaper than Au and Ag, respectively), abundancy, ease of manufacture, compatibility with optoelectronic devices and complementary metal–oxide–semiconductor (CMOS) technology, and it has material properties that enable plasmon resonances in a broad optical band [20,21]. The application of Al nanoplasmonics to label-free biosensing has been demonstrated recently [22,23,24]. Although Al may present oxidation and material degradation (corrosion and pitting) issues when exposed to aqueous solutions, which are particularly critical when thin Al films are concerned, these can be circumvented by depositing a thin protecting layer by atomic layer deposition [25] or through an oxygen passivation process. By using the latter technique, label-free optical biosensors based on Al nanohole arrays have been reported [22,23]; however, these were just proof-of-concept demonstrations and important issues such as sensor performance optimization and mass production capability were not studied in detail. 

Nanophotonic biosensor design is a mandatory step towards actual fabrication and commercialization of these devices. The design process should target both optimization of the sensor performance and facilitation of cost-effective manufacturability. The strong interaction of design factors and fabrication limitations makes the development and application of a proper design methodology necessary. Relevant reports on this issue, applied to refractometric 1D photonic sensors, can be found in [26,27]. Here, a design process of an Al-based multilayered 2D plasmonic crystal for label-free biosensing is presented. The device is formed of Al nanohole and nanodisk array layers separated by a polymeric film. The study of this structure is motivated by the successful demonstrations of Refs. [22,23], the use of cost-effective materials (Al and polymer), and the possibility of mass fabrication using nanoimprint lithography. The optical response of the device is calculated using numerical simulations. Resonance features exhibited by the configuration are analyzed in order to discover the role of the nanoscaled metal regions and their dimensions on the sensor response. Then, key biosensing performance functions are defined and calculated as a function of design dimensional parameters with the purpose of obtaining the desired sensor performance through a multi-objective desirability function. Finally, the effect of Al oxidation on the modeled sensor performance is examined.

## 2. Sensor Structure and Modeling

**Figure 1:**
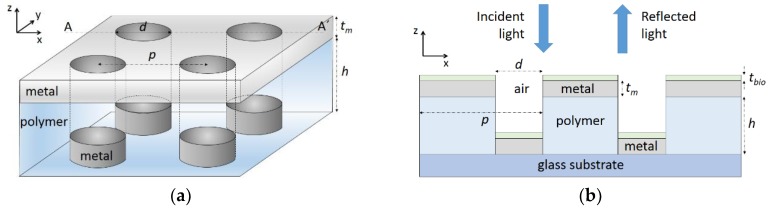
(**a**) Schematics of the modeled Al/polymer multilayered square lattice plasmonic crystal label-free biosensor; (**b**) Cross-sectional view along AA´ including a biolayer of thickness *t*_bio_ adhered on Al metal surfaces. Light along the −*z* axis impinges on the device, and the spectral distribution of normally reflected light is computed.

Figure 1 shows schematic diagrams of the studied configuration. The device consists of top and bottom Al layers of periodic patterns separated by a polymer layer. The top metal layer lies on the polymer layer, and both layers contain a coincident 2D nanohole square lattice. The bottom metal layer is a 2D disk square lattice located at the bottom of the polymer layer nanoholes. The structure rests on a silica glass substrate. A layer of thickness *t*_bio_, representing a biomolecule film, is assumed to be adhered on the Al surfaces, as shown in Figure 1b. The background material is air; that is, the sensor is considered to be operated in a dry state. The array period and Al metal thickness are denoted *p* and *t*_m_, respectively. The Al disk and nanohole diameters are equal and tagged as *d*. The polymer layer thickness (nanohole depth) is referred to as *h*.

Reflection spectrum and electric (E) field distributions at normal incidence were calculated using the finite-difference time-domain (FDTD) algorithm (FullWave, Rsoft). Periodic boundary conditions were chosen along the device plane coordinates (*x* and *y* axes of the array) and a perfectly matched layer (PML) boundary condition was used along the incident beam propagation direction (*z* axis). Frequency analysis of the reflection was achieved by launching a pulsed excitation from the superstrate region and calculating the fast Fourier transform (FFT) of the time-domain field component E_x_ of the reflected light on a plane above the device. The dielectric constant of Al was modeled using the well-known Drude–Lorentz equation with the fitted parameters reported elsewhere [28]. The considered refractive indices of the polymer layer and biolayer were 1.5 and 1.45, respectively, whereas those of air and the glass substrate were 1 and 1.523, respectively.

## 3. Design Parameters and Performance Functions

Device optimization targets both finding structural parameters that maximize the biosensor performance and minimizing the effects of variations of dimensional parameters. The latter is related to fabrication issues. In short, the described structure is envisaged to be fabricated as follows: first, a polymeric layer is spin-coated on a glass substrate; then, nanoimprint lithography is used to pattern the 2D nanohole square lattice on the polymer film, which may require a subsequent dry etching process to remove polymer residues; finally, an Al metal layer is deposited by thermal evaporation. The 2D crystal period and metal thickness can be defined by lithography and metal deposition, respectively, with high accuracy. On the other hand, the nanohole dimensions *h* (set by spin-coating) and *d* (determined by the nanoimprint mold and residue polymer removal) are expected to present poorer dimensional accuracies and repeatability. Thus, in our analysis, the plasmonic crystal period (*p*) and Al metal thickness (*t*_m_) were used as fixed parameters, whereas the nanohole diameter (*d*) and depth (*h*) were considered design parameters. 

We chose as fixed values *p* = 500 nm and *t*_m_ = 100 nm because these were used in actual Al nanohole array biosensors [22,23]. As will be seen in the next section, *p* largely determines the spectral position of the monitored plasmonic resonance; therefore, other values of *p* could be selected by the designer according to the read-out instrumentation. Both *d* and *h* were assumed to range from 100 nm to 300 nm with increments of 10 nm, which are typical values found in the fabrication of these types of configurations by conventional nanolithography. Dimensions smaller than 100 nm are difficult to achieve and control by top-down mass-production techniques, whereas polymer film thicknesses and nanohole diameters larger than 300 nm may require difficult-to-fabricate high-aspect-ratio nanoimprint molds and lead to inefficient surface plasmon excitation, respectively. 

The sensor response is defined as the spectral shift of a particular resonance feature due to biolayer thickness variation. As a consequence, the surface sensitivity (*S*_S_) of the device is given by *S*s = Δ*λ*/Δ*t*_bio_, where Δλ is the resonance wavelength shift and Δ*t*_bio_ is the biolayer thickness variation. The full width at half maximum (FWHM) of the resonance spectral feature is inversely related to the sensor resolution; therefore, a figure of merit (FOM) was defined as FOM = *S*s/FWHM [29]. In addition, signal noise can hinder the precise determination of a small-amplitude resonance; hence, large resonance amplitude is also desirable. Consequently, design optimization was carried out using *S*_S_, FOM, and resonance amplitude as biosensor performance functions. 

## 4. Results

### 4.1. Sensor Response and Analysis of Resonances

**Figure 2:**
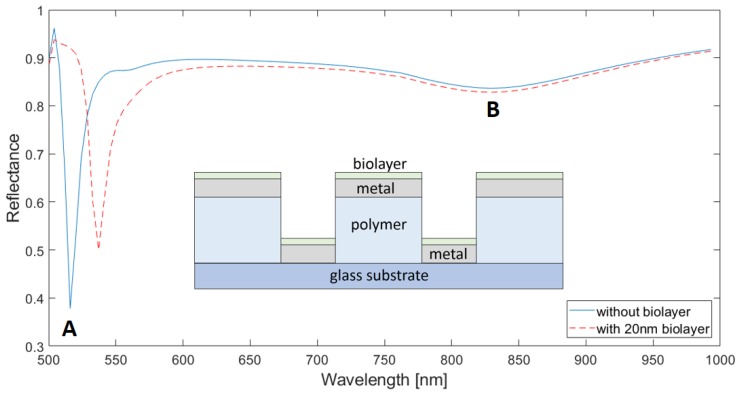
Reflectance spectra of a 500 nm period Al/polymer plasmonic 2D crystal (inset) for *d* = 150 nm and *h* = 150 nm. Al metal thickness is 100 nm. Blue and red (dotted) lines correspond to *t*_bio_ = 0 and *t*_bio_ = 20 nm, respectively. A and B indicate minima (dips) of the curve for t_bio_ = 0.

**Figure 3:**
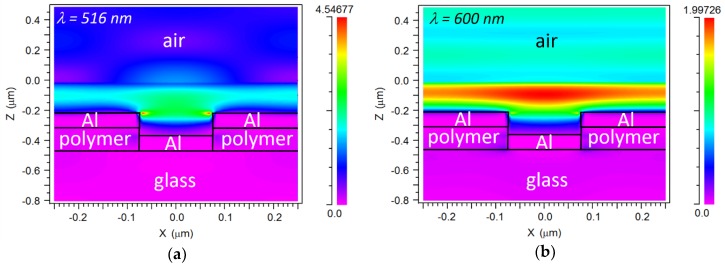

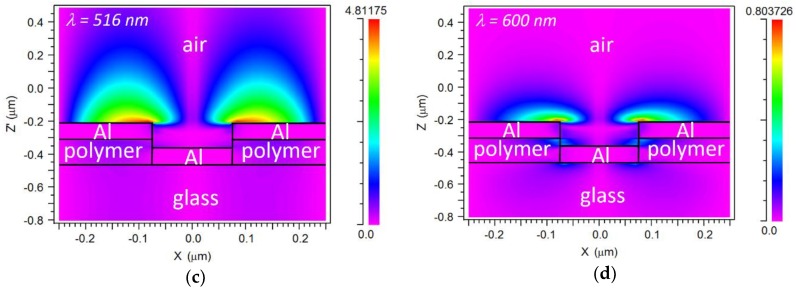
Cross-sectional electric field distributions of the studied configuration for *d* = 150 nm and *h* = 150 nm. (**a**) E_x_ at resonance (*λ* = 516 nm), (**b**) E_x_ out-of-resonance (*λ* = 600 nm), (**c**) E_z_ at resonance (*λ* = 516 nm), (**d**) E_z_ out-of-resonance (*λ* = 600 nm).

Figure 2 shows representative spectral reflectances of the studied sensor configuration, calculated for *d* = 150 nm and *h* = 150 nm. Blue and red lines correspond to the cases without an adhered biolayer (*t*_bio_ = 0) and with a 20 nm thick biolayer, respectively. For the no-biolayer case, dips are observed at *λ*_A_ = 516 nm and *λ*_B_ = 840 nm, the former being significantly deeper than the latter. According to the SPP grating coupling equation,

(1) $$\lambda_{SPP}\approx \frac{p}{\sqrt{i^{2}+j^{2}}}\sqrt{\frac{\varepsilon_{d}\varepsilon_{m}}{\varepsilon_{d}+\varepsilon_{m}}}$$

where *λ_SPP_* is a SPP resonance wavelength, *p* is the array period; *i* and *j* are grating orders and *ε_m_* and *ε_d_* are the dielectric functions of the metal and dielectric medium, respectively; minima A and B can be attributed to SPP resonances at the metal/air interface for *i* = ±1, *j* = 0 (or vice versa) and at the metal/polymer interface for *i* = ±1, *j* = 0 (or vice versa), respectively. Figure 3a,b (Figure 3c,d) shows E_x_-field (E_z_-field) cross-sectional distributions of the device at *λ*_A_ = 516 nm and *λ* = 600 nm (a nonresonant wavelength), respectively. Both E_x_ and E_z_ are enhanced at the metal/air interface for *λ*_A_ = 516 nm as compared to for *λ* = 600 nm, supporting the existence of an SPP resonance at the former wavelength. As predicted by Equation (1), it can be seen in Figure 2 that *λ*_A_-resonance red-shifts when a biolayer (with a higher dielectric function than that of air) is adhered to the device. Therefore, the *λ*_A_-resonance spectral variation can be used as the label-free biosensor response. Note also in Figure 2 that *λ*_B_ does not shift with the presence of the biolayer; this is because the calculated E_x_-field associated to that metal/polymer SPP resonance (not shown) does not interact with the biolayer significantly. 

**Figure 4:**
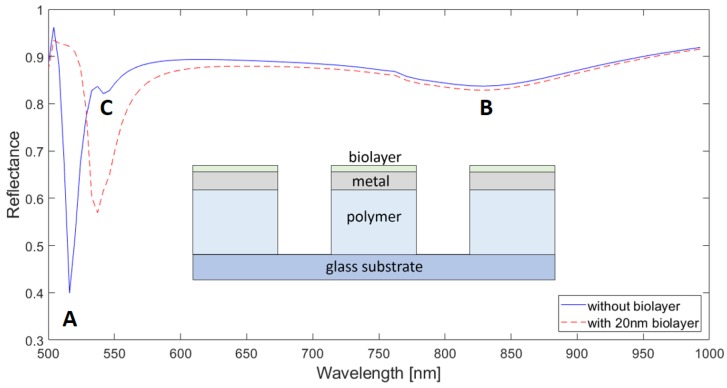
Reflectance spectra of a 500 nm period Al/polymer plasmonic 2D crystal without Al disks (inset) for *d* = 150 nm and *h* = 150 nm. Al metal thickness is 100 nm. Blue and red (dotted) lines correspond to *t*_bio_ = 0 and *t*_bio_ = 20 nm, respectively. A, B, and C indicate minima (dips) of the curve for *t*_bio_ = 0.

**Figure 5:**
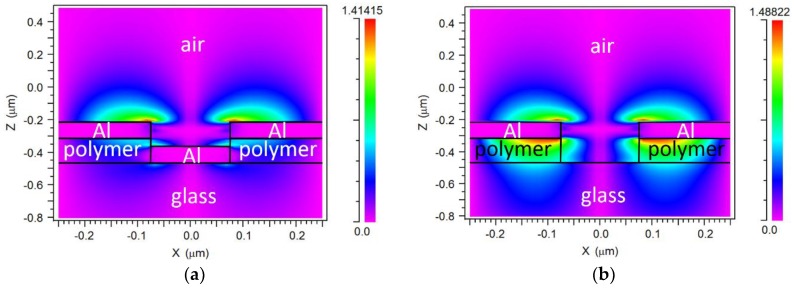
Cross-sectional electric (E_z_) field distributions of (**a**) the original configuration and (**b**) no-disk configuration for *d* = 150 nm and *h* = 150 nm at *λ* = 545 nm.

Equation (1) also predicts an SPP resonance for *i* = ±1, *j* = ±1 at the metal/polymer interface at 553 nm for the no-biolayer case, but no reflectance dip that could be associated with it is observed in Figure 2. To gain insight into this issue, we calculated the response of the original configuration (Figure 1) without the bottom Al disks (hereafter, no-disk configuration); this is plotted in Figure 4. In addition to the two dips displayed by the original structure (A and B), the no-disk configuration exhibits a reflectance minimum (C) at *λ*_C_ = 545 nm, which agrees well with the aforementioned (±1, ±1) metal/polymer SPP resonance. This suggests that the Al disks are responsible for the suppression of the (±1, ±1) metal/polymer SPP resonance in the original configuration. We attribute this effect to the overlap of the corresponding optical field with the lossy Al disk. This is corroborated by Figure 5a,b that plot the E_z_-field distributions of the original structure and no-disk configuration, respectively, at *λ*_C_ = 545 nm. Note that the high E-field at the Al/polymer interface shown in Figure 5b vanishes when the Al disks are present (Figure 5a). Both structures exhibit similar-intensity peaks at the metal/air interface because of the adjacent (±1,0) metal/air SPP resonance, which occurs at the same wavelength and with the same amplitude for both configurations (Figure 2 and Figure 4). Such metal/air SPP is not affected by the presence of the Al disk and the polymer film. 

From the biosensor performance point of view, the suppression of the (±1, ±1) metal/polymer SPP resonance is remarkably useful. Unlike the *λ*_A_-resonance, the (±1, ±1) metal/polymer SPP resonance is lowly sensitive to the presence of the biolayer. Therefore, since both resonances are spectrally close, when a biolayer is present, *λ*_A_-resonance red-shifts and overlaps the adjacent (±1, ±1) metal/polymer SPP resonance curve, as seen in Figure 4. Such an overlap increases the spectral width of the monitored *λ*_A_-resonance feature, degrading the biosensor performance in terms of resolution. The red curves in Figure 2 and Figure 4—which plot responses of the original and no-disk configurations, respectively, to the presence of a 20 nm thick biolayer—illustrate this result: the FWHM of the former is 15 nm, whereas the latter has a FWHM of 24 nm (for the sake of clarity, Figure S1 shows the performance of both configurations in the same graph). 

Our simulations revealed that the original configuration exhibits the (±1, ±1) metal/polymer SPP resonance when the nanohole dimensions are increased. This is attributed to a lower interaction of the resonance E-field with the Al disks, diminishing the attenuating effect of the latter. In particular, we noted that the (±1, ±1) metal/polymer SPP resonance appeared in the response of the original configuration for *d*, *h* ≥ 200 nm. Since such a metal/polymer SPP resonance is not desired, this imposes an upper limit to the design parameters. Therefore, we focused our design optimization on the dimensional range *d*, *h* ∈ [100 nm, 190 nm].

For the sake of completeness, it should be mentioned that SPP-related spectral features were also found in transmission, as shown in Figure S2 (Supplementary Data). However, unlike the reflectance dips, the calculated transmittance peaks were very low; in particular, that coinciding in wavelength with the reflectance dip A was two orders of magnitude smaller than the reflectance. This indicates that the reflectance dip A is mainly associated to light extinction. Low transmittance amplitude also justifies the use of reflection, instead of transmission, to interrogate the proposed nanostructure. 

### 4.2. Dimensions Optimization

**Figure 6:**
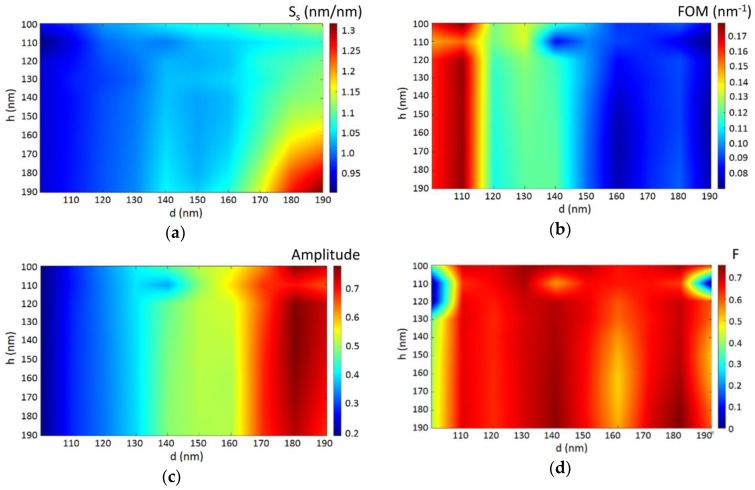
(**a**) Surface sensitivity (S_S_) in nm/nm; (**b**) figure of merit (FOM) in nm^−1^; (**c**) reflectance resonance amplitude and (**d**) desirability function (F) for *w*_1_ = 0.3, *w*_2_ = 0.5, and *w*_3_ = 0.3 of the studied Al/polymer 2D plasmonic crystal as a function of the design parameters *d* and *h*.

According to the previous analysis, *λ*_A_-resonance was used to define the performance functions introduced in Section 3. Figure 6a–c plot the calculated S_S_, FOM, and resonance amplitude, respectively, as a function of *d* and *h*. It is seen that S_S_ increases as both *h* and *d* increase. This is because, for the no-biolayer case, *λ*_A_ red-shifts as the nanohole dimensions increase; it is well known that for SP-based refractometric sensors, the longer the operation wavelength, the larger the spectral shift associated to a particular refractive index change of the interrogated sample. It is also observed that both the FOM and dip amplitude have low sensitivity to *h* and exhibit opposite trends with *d* variation. For better understanding of the FOM results, the calculated FWHM plot has been included in Supplementary Data (Figure S3). Note, in addition, the presence of a sharp change at *h* = 110 nm for all *d* values. We attribute this to optical coupling to the polymer layer produced by the resulting 10 nm nanogap between the top metal layer and the metal disk [30,31]. Light leaked to the polymer layer may affect the field intensity associated with the metal/air SPR (peak A) and, therefore, to the surface sensitivity and the resonance dip amplitude. For *h* = 100 nm, there is no metal nanogap, whereas for *h* = 120 nm, the nanogap may be too large to favor the aforementioned optical coupling. In any case, there is no improvement of the device performance for *h* = 110 nm, and, therefore, this effect has not been studied in detail in this work. 

Since the three performance functions behave differently, we used a desirability function [32] to optimize the device parameters in terms of both performance and fabrication feasibility. Large values are desirable for the three performance functions; therefore, we can define individual desirability functions D1, D2, and D3 for S_S_, FOM, and amplitude, respectively, as

(2) $$D_{i}={\left(\frac{V_{i}-m_{i}}{M_{i}-m_{i}}\right)}^{w_{i}}$$

where *i* = 1, 2, or 3; *V_i_*, *M_i_*, and *m_i_* are the actual, maximum, and minimum values of the considered performance function within the design parameter dimensional range; and *w_i_* is the relative weight (0 ≤ *w_i_* ≤ 1). Thus, the overall desirability function *F* can be calculated as

(3) $$F={\left(D_{1}\times D_{2}\times D_{3}\right)}^{1/3}.$$

Depending on the required specifications, the designer can emphasize the importance of each performance function by setting proper values for the weight factors. For example, if FOM is decided to be more important than S_S_ and dip amplitude, a suitable selection of values could be *w*_1_ = 0.3, *w*_2_ = 0.5, and *w*_3_ = 0.3. For this case, the resulting desirability function is plotted in Figure 6d. Three *F*-maximum (*F* ≈ 0.75) regions or bands lowly dependent on h are observed at *d* = 110, 150, and 180 nm. From a fabrication point of view, low *h*-sensitivity represents large tolerance to deviations from the targeted polymer layer thickness. This is a relevant result because significant resist thickness variations may arise from a spin-coating process. In particular, the maximum thickness tolerance (approximately ± 30 nm) is obtained for *h* = 150 nm. Among the three *F*-maximum bands, that centered at *d* = 140 nm shows the largest *d*-dimension tolerance: ± 10 nm. This tolerance is typically found in the fabrication of nanohole arrays by nanolithography techniques; it is therefore reasonable to target such a *d* value. Thus, the optimum nanohole dimensions, from performance and fabrication points of view, would be *h* = 150 nm and *d* = 140 nm. The corresponding performance functions for these dimensions are S_S_ = 1.04 nm/nm, FOM = 0.12 nm^−1^, and resonance dip amplitude = 0.48. For the sake of illustration, if the adhered biolayer is, for example, a monolayer of BSA (bovine serum albumin) antibodies (BSA antibody size ≈ 12 nm; BSA monolayer surface density ≈ 1.7 ng/mm^2^ [33]), then a surface sensitivity of 1.04 nm/nm is equivalent to a biomolecule mass surface concentration sensitivity of 7.3 nm/(ng/mm^2^). Thus, if the resolution of our measurement system were 0.3 nm (easily achieved by commercial spectrometers), this would mean a limit of detection (LOD) of 41 pg/mm^2^, which is of the same order of magnitude to that exhibited by other label-free optical nanobiosensors [33]. Note also that the obtained optimum *h* and *d* values indicate a nanohole aspect ratio of ~1, which is a modest value that should facilitate the fabrication of nanoimprint molds by conventional dry etching techniques. 

The purpose of function *F* is to assist the designer in the choice of the optimum design parameters according to specifications. Different values of *w_i_* can be selected even for the same scenario. Therefore, the designer must check the particular values of the performance functions (S_S_, FOM, and amplitude) resulting from each selection of *w_i_*, and vary them accordingly in case they do not meet specifications or require the implementation of unfeasible nanofeature dimensions.

### 4.3. Effect of Al Oxidation

**Figure 7:**
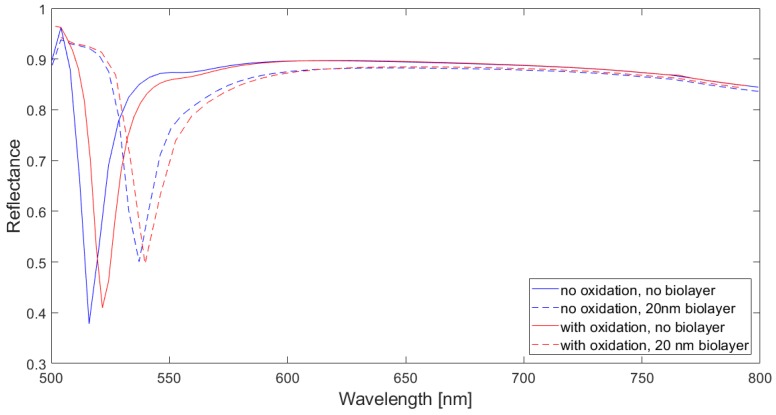
Reflectance spectra of a 500 nm period Al/polymer plasmonic 2D crystal (*d* = 150 nm, *h* = 150 nm) without (blue curves) and with (red curves) a 5 nm thick Al oxide layer. Solid and dashed lines correspond to the absence or presence of a 20 nm thick biolayer, respectively.

We also analyzed the effect of Al oxidation since Al naturally forms a thin surface layer of aluminum oxide in contact with air. Figure 7 shows the computed spectral reflectivity of the studied configuration (*d* = 150 nm, *h* = 150 nm) when a 5 nm thick oxide layer (Al oxide refractive index = 1.77) is assumed to cover the Al regions conformally. It is seen that the response of the device slightly differs from that of the unoxidized device. As a consequence of the presence of the oxide layer, the metal/air SPP resonance wavelength red-shifts. The calculated surface sensitivity, FOM, and dip resonance amplitude for the oxidized configuration were 0.94 nm/nm, 0.08 nm^−1^, and 0.48, respectively, whereas those of its unoxidized counterpart were S_S_ = 1.02 nm/nm, FOM = 0.09 nm^−1^, and dip amplitude = 0.5. This indicates that the effect of Al surface oxidation on the sensor performance is not significant. In addition, such a thin aluminum oxide layer can act as a physical barrier to further oxidation in aqueous solutions [22], which supports the applicability of the analyzed device to actual biological tests. 

## 5. Discussion

The presented design methodology was inspired by that reported by Ju et al. [27] for 1D dielectric grating label-free biosensors. There are, however, important differences between both works that arise from the different device architectures, performances and operation conditions. For example, regarding device performance functions, the ratio of bulk sensitivity to surface sensitivity was selected as an objective function in [27] because that sensor was planned to operate in aqueous solution. Since our device is intended to be used in dry conditions, we did not study such a ratio. We decided to operate the biosensor in air because it should be more sensitive in this medium than immersed in a liquid: refractive index changes caused by molecular adsorption are larger in air than in liquids [10]. Additionally, operation in air is a feasible and practical procedure when either sensor architecture simplicity (thus avoiding microfluidic integration) or prototyping is targeted [10,33,34], or/and the effect of a liquid background is not desired. Operation in air is also known as “dip-and-dry” operation mode [35] because it typically involves chip incubation in the solution of interest and subsequent chip drying by a gentle stream of nitrogen. 

Another performance function contemplated in [27] was the FWHM because of the extremely narrow resonance peaks exhibited by the analyzed 1D photonic crystal, which could make the spectral resolution of the measurement system a limiting factor. In our case, we have considered that the measurement of the FWHM is not an issue because this value is typically larger than a few nanometers for the studied configuration and can be easily measured by cost-effective compact spectrometers. Instead, we have determined the resonance amplitude to be a more relevant performance function because this value depends on the design parameter *d*, and it becomes significantly reduced as *d* is decreased. Too small resonance amplitude may make it extremely difficult to measure the monitored resonance wavelength in the presence of noise.

It should be mentioned that in an actual device the biofilm could also conformally coat the sidewalls of the Al nanoholes. We carried out simulations assuming such a situation and found no significant deviations from the results presented in this work (see Supplementary Data, Figure S4). That is, the contribution of the sidewall biofilm effect to the overall sensor performance is negligible. Since simulations with the sidewall biofilm regions required more computation time than those without it, we used the model shown in Figure 1b.

There are several reported Al plasmonic 1D grating configurations that exhibit better performances than the structure analyzed here. These are mainly based on Fano resonances [36], which require interrogating the structures at specific angles, and metal gratings with nanocavities [37], which contain nanostrip widths on the order of nanometers that are difficult to fabricate with high accuracy. Note also that those 1D periodic configurations are highly polarization sensitive and, therefore, require the use of polarizers or polarized light sources, which increases the cost and complexity of the read-out system. In any case, the main goal of this work is not to improve the performance of those devices, but to analyze and to provide a design methodology for the proposed configuration as a first step towards its fabrication by nanoimprint lithography and subsequent normal-incidence optical interrogation. We also expect that the design procedure used for this particular structure, based on the guidelines

(a) selection of design parameters,
(b) selection of performance functions,
(c) analysis of resonances, and
(d) design optimization,

could be extended to the design of other planar plasmonic crystal configurations for label-free biosensing.

Finally, we remark upon the positive effect on the sensor response (suppression of an undesired resonance) produced by the presence of the Al disks. This result indicates the convenience of having metal disks in the studied biosensing scheme versus using a single metal nanohole array layer as in [22,23]. Note that the incorporation of metal disks does not imply a specific or additional fabrication process as they are naturally formed when the metal film is deposited on the nanopatterned polymer layer. 

## 6. Conclusions

We have analyzed and designed an Al 2D plasmonic nanocrystal label-free biosensor. The performance of the modeled device, consisting of Al nanohole and nanodisk array films separated by a polymer layer, was simulated using the FDTD method. The device exhibits a clear SPP resonance sensitive to the thickness of an adhered biolayer and, therefore, is appropriate for use as the label-free sensor response. Simulations also showed that the Al disks avoid the appearance of an SPP at the metal/polymer interface adjacent to the monitored biolayer-sensitive SPP resonance. This effect prevents the overlapping between both resonances, improving the sensor resolution. Nanohole dimensions were used as design parameters and optimized according to a multi-objective desirability function that allows the sensor designer to weigh the importance of key performance functions. Calculations indicated that the effect of spontaneous Al oxidation should not affect the sensor performance significantly. The presented design method is expected to be valuable to nanophotonic biosensor researchers and engineers for implementing marketable devices according to the desired specifications and available technology.

## Supplementary Materials

The following are available online at http://www.mdpi.com/1424-8220/18/10/3335/s1, Figure S1: Calculated reflectance spectra for t_bio_ = 20 nm of 500 nm period Al/polymer plasmonic 2D crystals (d = 150 nm, h = 150 nm) with Al disks (red curve) and without Al disks (blue curve). Figure S2: Calculated transmittance spectrum of a 500 nm period Al/polymer plasmonic 2D crystal for d = 150 nm and h = 150 nm. Transmittance values are two orders of magnitude smaller than reflectance values (Figure 2). Figure S3: FWHM in nm of the studied Al/polymer 2D plasmonic crystal as a function of the design parameters d and h. Figure S4: Reflectance spectra of a 500 nm period Al/polymer plasmonic 2D crystal (*d* = 150 nm, *h* = 150 nm) with (blue curve) a 20 nm thick biolayer conformally covering the nanohole Al sidewalls (inset) and without (red curve) such a biolayer sidewall region (Figure 1b). Biolayer sidewall contribution to the sensor performance is negligible.

## References

[1] Lindquist N.C., Nagpal P., McPeak K.M., Norris D.J., Oh S.-H. (2012). Engineering metallic nanostructures for plasmonics and nanophotonics. Rep. Prog. Phys..

[2] Masson J.F., Zhao S.S., Vestergaard M., Kerman K., Hsing I.M., Tamiya E. Plasmonic Sensors for Analysis of Proteins and an Oncologic Drug in Human Serum. Nanobiosensors and Nanobioanalyses.

[3] Gomez-Cruz J., Nair S., Manjarrez-Hernández A., Gavilanes-Parra S., Ascanio G., Escobedo C. (2018). Cost-effective flow-through nanohole array-based biosensing platform for the label-free detection of uropathogenic *E. coli* in real time. Biosens. Bioelectron..

[4] Raschke G., Kowarik S., Franzl, Sönnichsen T., Klar T.A., Feldmann J. (2003). Biomolecular recognition based on single gold nanoparticle light scattering. Nano Lett..

[5] Sakono M., Zako T., Maeda M. (2012). Naked-eye detection of amyloid aggregates using gold nanoparticles modified with amyloid beta antibody. Anal. Sci..

[6] Kneipp K., Kneipp H., Itzkan I., Dasari R.R., Feld M.S. (1999). Ultrasensitive chemical analysis by Raman spectroscopy. Chem. Rev..

[7] Brolo A.G., Gordon R., Leathem B., Kavanagh K.L. (2004). Surface plasmon sensor based on the enhanced light transmission through arrays of nanoholes in gold films. Langmuir.

[8] De Leebeeck A., Kumar L.K., de Lange V., Sinton D., Gordon R., Brolo A.G. (2007). On-Chip Surface-Based Detection with Nanohole Arrays. Anal. Chem..

[9] Zhao J., Zhang X., Yonzon C.R., Haes A.J., van Duyne R.P. (2006). Localized surface plasmon resonance biosensors. Nanomedicine.

[10] Shen Y., Zhou J., Liu T., Tao Y., Jiang R., Liu M., Xiao G., Zhu J., Zhou Z.-K., Wang X. (2013). Plasmonic gold mushroom arrays with refractive index sensing figures of merit approaching the theoretical limit. Nature Commun..

[11] Sharpe J.C., Mitchell J.S., Lin L., Sedoglavich N., Blaikie R.J. (2008). Gold nanohole array substrates as immunobiosensors. Anal. Chem..

[12] Artar A., Yanik A.A., Altug H. (2009). Fabry–Pérot nanocavities in multilayered plasmonic crystals for enhanced biosensing. Appl. Phys. Lett..

[13] Yang J.-C., Ji J., Hogle J.M., Larson D.N. (2009). Multiplexed plasmonic sensing based on small-dimension nanohole arrays and intensity interrogation. Biosens. Bioelectron..

[14] Escobedo C. (2013). On-chip nanohole array based sensing: A review. Lab Chip.

[15] Pilát Z., Kizovský M., Ježek J., Krátký S., Sobota J., Šiler M., Samek O., Buryška T., Vaňáček P., Damborský J. (2018). Detection of Chloroalkanes by Surface-Enhanced Raman Spectroscopy in Microfluidic Chips. Sensors.

[16] Sinton D., Gordon R., Brolo A.G. (2008). Nanohole arrays in metal films as optofluidic elements: Progress and potential. Microfluid. Nanofluid..

[17] Yang J.-C., Ji J., Hogle J.M., Larson D.N. (2008). Metallic Nanohole Arrays on Fluoropolymer Substrates as Small Label-Free Real-Time Bioprobes. Nano Lett..

[18] Chuo Y., Hohertz D., Landrock C., Omrane B., Kavanagh K.L., Kaminska B. (2013). Large-Area Low-Cost Flexible Plastic Nanohole Arrays for Integrated Bio-Chemical Sensing. IEEE Sens. J..

[19] Polyanskiy M.N. Refractive Index Database. https://refractiveindex.info/.

[20] Martin J., Kociak M., Mahfoud Z., Proust J., Gerard D., Plain J. (2014). High-resolution imaging and spectroscopy of multipolar plasmonic resonances in aluminum nanoantennas. Nano Lett..

[21] Sobhani A., Manjavacas A., Cao Y., McClain M.J., de Abajo J.F., Nordlander P., Halas N.J. (2015). Pronounced linewidth narrowing of an aluminum nanoparticle metallic film. Nano Lett..

[22] Canalejas-Tejero V., Herranz S., Bellingham A., Moreno-Bondi M.C., Barrios C.A. (2014). Passivated Aluminum Nanohole Arrays for Label-Free Biosensing Applications. ACS Appl. Mater. Interfaces.

[23] Barrios C.A., Canalejas-Tejero V., Herranz S., Moreno-Bondi M.C., Avella-Oliver M., Puchades R., Maquieira A. (2014). Aluminum nanohole arrays fabricated on polycarbonate for compact disc-based label-free optical biosensing. Plasmonics.

[24] Li W., Qiu Y., Zhang L., Jiang L., Zhou Z., Chen H., Zhou J. (2016). Aluminum nanopyramid array with tunable ultraviolet–visible–infrared wavelength plasmon resonances for rapid detection of carbohydrate antigen 199. Biosens. Bioelectron..

[25] Zhang X., Zhao J., Whitney A.V., Elam J.W., Van Duyne R.P. (2006). Ultrastable substrates for surface-enhanced Raman spectroscopy: Al2O3 overlayers fabricated by atomic layer deposition yield improved anthrax biomarker detection. J. Am. Chem. Soc..

[26] Block I., Ganesh N., Lu M., Cunningham B.T. (2008). A sensitivity model for predicting photonic crystal biosensor performance. IEEE Sens. J..

[27] Ju J., Han Y.-A., Kim S.-M. (2013). Design Optimization of Structural Parameters for Highly Sensitive Photonic Crystal Label-Free Biosensors. Sensors.

[28] Rodrigo S., García-Vidal F., Martín-Moreno L. (2008). Influence of material propertieson extraordinary optical transmission through hole arrays. Phys. Rev. B Condens. Matter.

[29] Otte M.A., Sepúlveda B., Ni W., Pérez Juste J., Liz-Marzán L.M., Lechuga L.M. (2010). Identification of the optimal spectral region for plasmonic and nanoplasmonic sensing. ACS Nano.

[30] Kim M.-K. (2015). Efficient coupling of a sub-5-nm-gap plasmonic crystal cavity with an integrated waveguide. Opt. Express.

[31] Eggleston M.S., Wu M.C. (2015). Efficient Coupling of an Antenna-Enhanced nanoLED into an Integrated InP Waveguide. Nano Lett..

[32] Montgomery D. (2001). Design and Analysis of Experiments.

[33] Barrios C.A., Bañuls M.J., González-Pedro V., Gylfason K.B., Sánchez B., Griol A., Maquieira A., Sohlström H., Holgado M., Casquel R. (2008). Label-free optical biosensing with slot-waveguides. Opt. Lett..

[34] Sanza F.J., Holgado M., Ortega F.J., Casquel R., López-Romero D., Bañuls M.J., Laguna M.F., Barrios C.A., Puchades R., Maquieira A. (2011). Bio-Photonic Sensing Cells over transparent substrates for anti-gestrinone antibodies biosensing. Biosens. Bioelectron..

[35] Banica F.-G. (2012). Chemical Sensors and Biosensors Fundamentals and Applications.

[36] Lee K.-L., Hsu H.-Y., You M.-L., Chang C.-C., Pan M.-Y., Shi X., Ueno K., Misawa H., Wei P.-K. (2017). Highly Sensitive Aluminum-Based Biosensors using Tailorable Fano Resonances in Capped Nanostructures. Sci. Rep..

[37] Lu X., Lin J. (2017). Field enhancement of a metal grating with nanocavities and its sensing applications. J. Opt..

