# Supplementary material for: Design of an Aluminum/Polymer Plasmonic 2D Crystal for Label-Free Optical Biosensing
Source: Sensors (Basel). 2018 Oct 5;18(10):3335. doi: 10.3390/s18103335 (PMC6211116; doi:10.3390/s18103335)
Supplement: Supplementary file 1 [file sensors-18-03335-s001.pdf]

Article

Design of an Aluminum/Polymer Plasmonic 2D Crystal for Label-Free Optical Biosensing

Luca Tramarin [1] and Carlos Angulo Barrios [1,2,*]

1 Instituto de Sistemas Optoelectrónicos y Microtecnología (ISOM), ETSI Telecomunicación, Universidad Politécnica de Madrid, Ciudad Universitaria s/n, Madrid 28040, Spain; luca.tramarin@alumnos.upm.es
2 Department of Photonics and Bioengineering (TFB), ETSI Telecomunicación, Universidad Politécnica de Madrid, Ciudad Universitaria s/n, Madrid 28040, Spain
* Correspondence: carlos.angulo.barrios@upm.es; Tel.: +34-91-549-5700

Supplementary Data

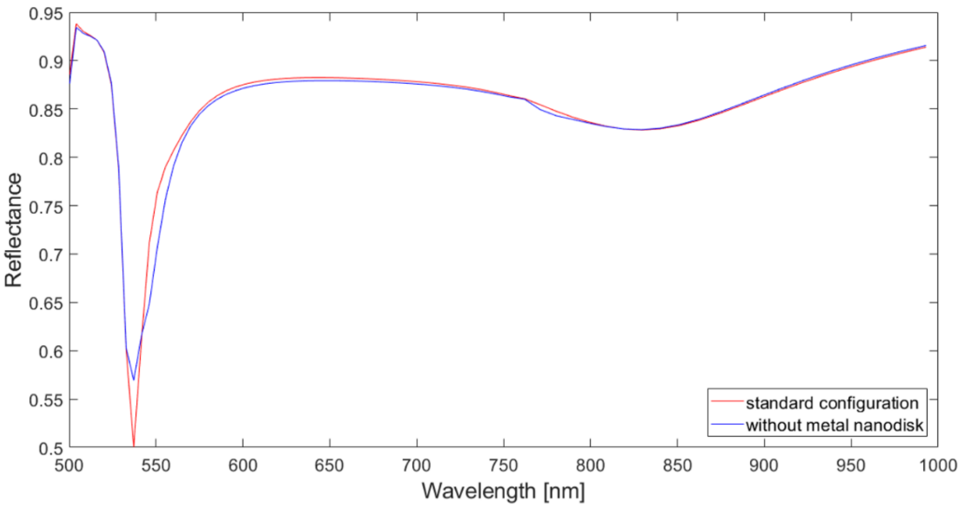

Figure S1. Calculated reflectance spectra for t_{bio} = 20 nm of 500 nm period Al/polymer plasmonic 2D crystals (*d* = 150 nm, *h* = 150 nm) with Al disks (red curve) and without Al disks (blue curve).

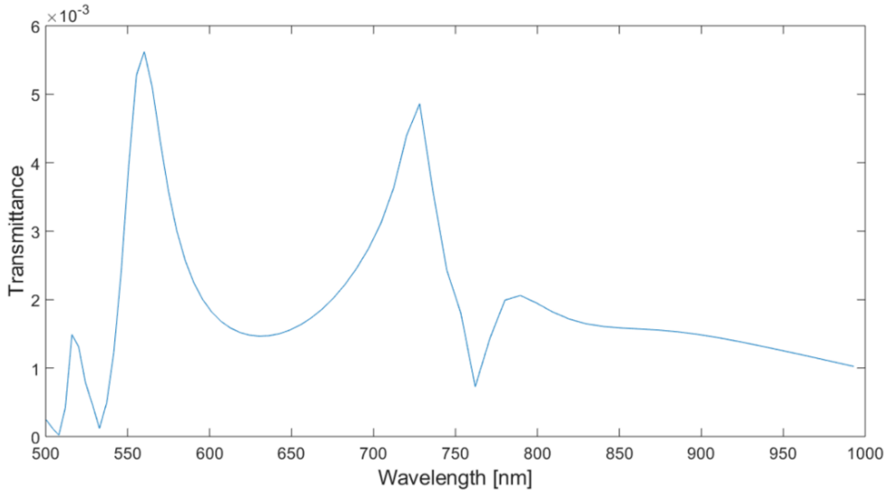

Figure S2. Calculated transmittance spectrum of a 500 nm period Al/polymer plasmonic 2D crystal for *d* = 150 nm and *h* = 150 nm. Transmittance values are two orders of magnitude smaller than reflectance values (Figure 2).

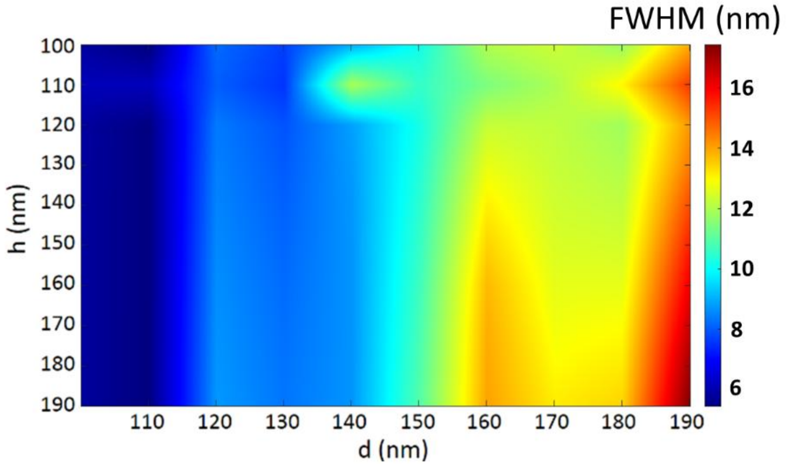

Figure S3. FWHM in nm of the studied Al/polymer 2D plasmonic crystal as a function of the design parameters *d* and *h*.

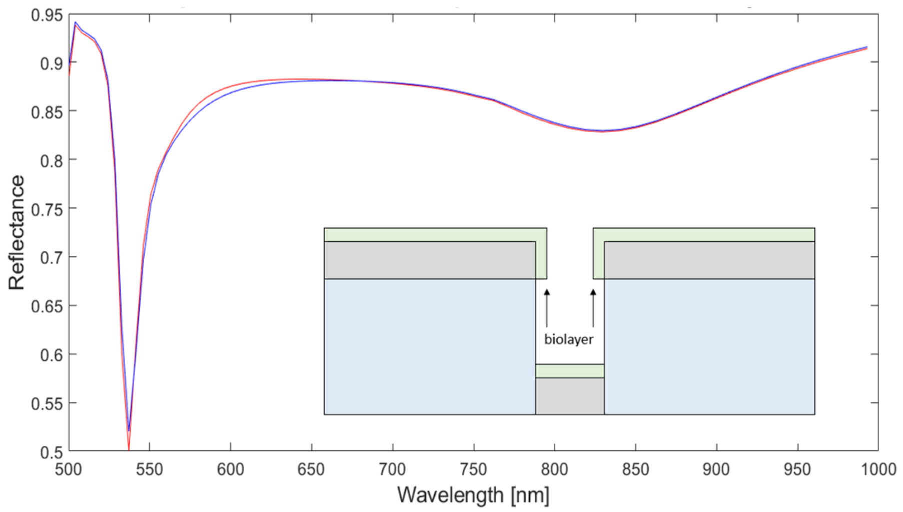

Figure S4. Reflectance spectra of a 500 nm period Al/polymer plasmonic 2D crystal (*d* = 150 nm, *h* = 150 nm) with (blue curve) a 20 nm thick biolayer conformally covering the nanohole Al sidewalls (inset) and without (red curve) such a biolayer sidewall region (Figure 1b). Biolayer sidewall contribution to the sensor performance is negligible.
